# Possible prevention of uremic nausea by vitamin D receptor activators in non-dialysis patients with stage 5 chronic kidney disease

**DOI:** 10.1007/s10157-016-1355-8

**Published:** 2016-11-14

**Authors:** Masato Ikeda, Yoshimi Ueda, Yukio Maruyama, Keitaro Yokoyama, Takashi Yokoo, Nobuhiko Joki, Ryoichi Ando, Toshio Shinoda, Daijo Inaguma, Toshihiko Yamaka, Yasuhiro Komatsu, Fumihiko Koiwa, Toshifumi Sakaguchi, Shigeo Negi, Takashi Shigematsu

**Affiliations:** 1 Division of Nephrology and Hypertension, The Jikei University School of Medicine Katsushika Medical Center, 6-41-2 Katsushika-ku, Tokyo, 125-8506 Japan; 2 Division of Nephrology and Hypertension, Department of Internal Medicine, The Jikei University School of Medicine, Tokyo, Japan; 3 Division of Nephrology, Toho University Ohashi Medical Center, Tokyo, Japan; 4 Department of Nephrology, Musashino Red Cross Hospital, Tokyo, Japan; 5 Dialysis Center, Kawakita General Hospital, Tokyo, Japan; 6 Kidney Center, Nagoya Daini Red Cross Hospital, Nagoya, Japan; 7 Division of Clinical Engineering, Department of Technology, Kanagawa Institute of Technology, Kanagawa, Japan; 8 Division of Internal Medicine, Department of Nephrology, St. Luke’s International Hospital, Tokyo, Japan; 9 Division of Nephrology, Division of Internal Medicine, Showa University Fujigaoka Hospital, Yokohama, Japan; 10 Division of Nephrology and Blood Purification Medicine, Wakayama Medical University, Wakayama, Japan

**Keywords:** Vitamin D receptor, Activator, Uremic, Nausea, Chronic kidney disease, Dialysis

## Abstract

**Background:**

Nausea is a major uremic symptom and a frequent indication for starting dialysis. However, preventive medication for uremic nausea has not yet been identified. Vitamin D receptor activators (VDRAs) may prevent uremic nausea via their pleiotropic actions. The objective of this study was to explore whether VDRA administration during the predialysis period is associated with a reduced prevalence of uremic nausea just prior to beginning dialysis.

**Methods:**

A multicenter, retrospective, cross-sectional study was performed to identify a medication to prevent uremic nausea. Patients with stage 5 CKD who were followed-up over 3 months were included. The primary outcomes examined were the prevalence of uremic nausea, congestive heart failure (CHF), and intractable edema at dialysis commencement. The predictor variable was VDRA use during the predialysis period.

**Results:**

One thousand five hundred and thirty six patients who had just begun dialysis in nine Japanese facilities between January 2006 and October 2013 were included. Two hundred and thirty (15.0%) patients had commenced dialysis because of uremic nausea, and three hundred and ninety two (25.5%) patients had been using VDRAs before initiating dialysis. Logistic regression analysis showed that, among the medications examined in this study, only VDRA use was independently associated with a lower frequency of uremic nausea (OR 0.512, 95% CI 0.347–0.738, *P* = 0.0003). On the other hand, CHF and intractable edema were not associated with VDRA administration.

**Conclusion:**

Use of VDRAs during the predialysis period was the only factor associated with a lower prevalence of uremic nausea, suggesting that VDRAs may prevent uremic nausea in patients with advanced CKD.

## Introduction

Uremic nausea is a common symptom in patients with advanced CKD, it increases in severity with the progression of CKD, especially below a creatinine clearance of 25 mL/min, and it may lead to protein-energy wasting, morbidity, and mortality [1, 2]. A large clinical trial involving 20,854 patients showed that the leading cause for dialysis commencement was uremic nausea, and the proportion of all patients at the initiation of dialysis with uremic nausea was 46.3% in Japan [3]. Predialysis patients with uremic nausea may regain their appetite soon after starting dialysis [4], presumably because of the removal of toxic molecules due to diffusive transport through hemodialysis membranes [5]. In this regard, uremic nausea in non-dialysis patients may differ from anorexia that appears in long-term maintenance dialysis patients. On the other hand, anorexia that appears in long-term maintenance dialysis patients does not usually disappear after each dialysis session. There are currently no medications to prevent uremic nausea in patients with advanced CKD. The objective was to explore which medication is associated with a reduced prevalence of dialysis-requiring uremic nausea in predialysis patients with stage 5 CKD. Vitamin D/vitamin D receptor activators (VDRAs) are among the candidate medications that may prevent uremic nausea. Vitamin D is a pleiotropic steroid hormone that has anti-inflammatory and immunomodulatory actions in various cells and tissues, besides regulating calcium, phosphate, and PTH pathways [6]. Recent studies have shown that 1, 25(OH)_2_D_3_ downregulates genes by blocking NF-κB activation in gastrointestinal cells [7], and the therapeutic role of vitamin D/VDRAs in clinical and experimental models of inflammatory bowel disease has been reported [8]. Anti-inflammatory actions of VDRAs have also been reported in both dialysis and non-dialysis patients with CKD [9, 10]. Thus, this study focused on VDRA use in the predialysis period, and its association with uremic nausea development was examined.

We have previously reported that use of VDRAs in predialysis patients with stage 5 CKD was not independently associated with reduction of the prevalence of dialysis-requiring congestive heart failure (CHF) [11]. However, the ability of VDRAs to prevent uremic nausea has not been assessed in patients with advanced CKD. To the best of our knowledge, this is the first report to examine the association between VDRA administration and uremic nausea development in the predialysis period.

## Methods

### Study design

This study used a cross-sectional, observational, multicenter design, and included 3447 Japanese patients across 9 Japanese institutions who initiated dialysis between January 1, 2006 and October 31, 2013. All institutions belonged to the Japanese Study Group for Assessing Initiation of Renal Replacement Therapy (JSTART), and these institutions served as the primary predialysis source of information for patients with end-stage kidney disease.

Clinical information and hematological data were collected at the institutional level immediately before the first hemodialysis session, according to the JSTART database sheet (Microsoft Excel). Each patient’s information was only labeled with the institution and patient number to protect the patients’ privacy. This study was performed in accordance with the Declaration of Helsinki. The ethics committee for clinical research of Jikei University School of Medicine approved this study [permission no. 25–343 (7849)].

**Fig. 1:**
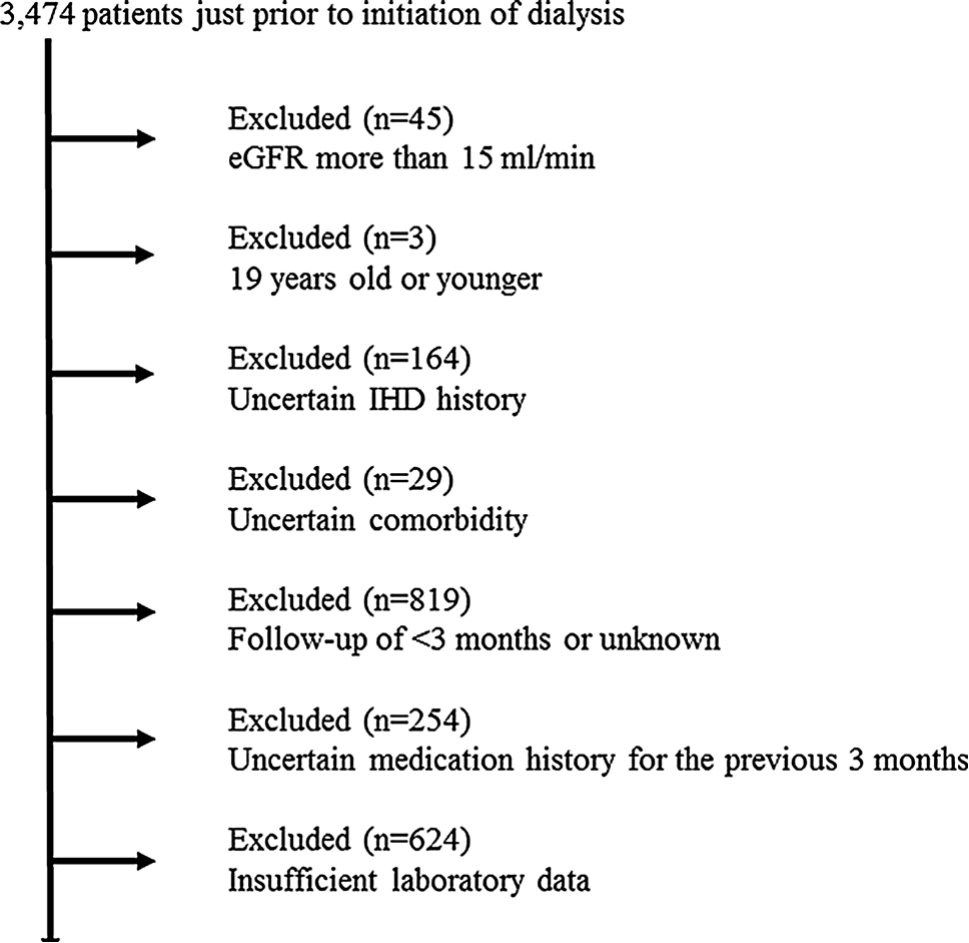
Flow chart of recruitment and screening of study participants. *eGFR* estimated glomerular filtration rate, *IHD* ischemic heart disease. One thousand nine hundred and eleven patients were excluded from the analysis due to having stage 1–4 CKD (45 patients), being ≤19 years old (3 patients), having an uncertain history of ischemic heart disease (IHD) (164 patients) and comorbidity (29 patients), a follow-up period of <3 months or unknown length (819 patients), lack of data regarding medication use in the previous 3 months (254 patients), and insufficient laboratory data (624 patients). Therefore, of the 3474 patients evaluated, 1536 Japanese patients satisfied the inclusion criteria and were included in the analysis

To explore the association between CKD medications and dialysis-requiring uremic nausea, the following inclusion criteria were selected: (1) only stage 5 CKD Japanese patients who were followed-up over 3 months by nephrologists to exclude emergent initiation of dialysis for acute kidney injury (AKI); and (2) patient records with complete data for the following factors: age, sex, presence of diabetic kidney disease, reasons (uremic symptoms) for initiating dialysis, history of ischemic heart disease, duration of nephrologist follow-up, systolic and diastolic blood pressures, and laboratory data [hemoglobin, albumin, urea nitrogen, creatinine, sodium, potassium, chloride, corrected calcium, phosphorus, C-reactive protein (CRP), and intact parathyroid hormone (intact PTH)]. As a result, 1911 patients were excluded from the analysis due to having stage 1–4 CKD (45 patients), being ≤19 years old (3 patients), having an uncertain history of ischemic heart disease (IHD) (164 patients) and comorbidity (29 patients), a follow-up period of <3 months or unknown length (819 patients), lack of data regarding medication use in the previous 3 months (254 patients), and insufficient laboratory data (624 patients). Therefore, of the 3474 patients evaluated, 1536 Japanese patients satisfied the inclusion criteria and were included in the analysis (Fig. 1).

The eGFR was calculated using the new Japanese equation [12]: eGFR (mL/min/1.73 m^2^) = 194 × Cr^−1.094^ × age^−0.287^ (×0.739 for women).

The reasons for initiating dialysis were classified into 5 categories in a similar manner to the national surveillance in Japan (3): CHF, uremic nausea, intractable edema, other symptoms (anemia, neurological, or other symptoms), and planned dialysis without uremic symptoms.

A diagnosis of uremic nausea was made based on the presence of nausea that developed along with exacerbation of kidney function without obvious gastrointestinal disease. The exact characteristic of uremic nausea was rapid resolution after the first hemodialysis session.

The criteria for a diagnosis of CHF have been reported previously [11]. Information regarding the patients’ medication use before starting dialysis was also collected. The specific medications that were evaluated were erythropoiesis-stimulating agents (ESAs), renin-angiotensin-aldosterone system (RAAS) inhibitors (ACEIs or ARBs), calcium channel blockers (CCBs), loop diuretics, other anti-hypertensive agents (alpha-blockers and/or beta-blockers and/or other hypertensive drugs), and VDRAs, calcium carbonate (CaCO_3_), and AST-120. AST-120 (Daiichi-Sankyo Industry Co., Tokyo, Japan) is a carbonaceous adsorbent that is used to treat patients with CKD.

### Variables

The primary outcomes were the prevalences of uremic nausea, CHF, and intractable edema at dialysis initiation, and the predictor variable was use of VDRAs during the predialysis period. The following explanatory variables were evaluated: sex; age; comorbid diabetic kidney disease; nephrologist follow-up period; use of loop diuretics, ESAs, or CaCO_3_; eGFR; serum corrected calcium; serum phosphorus; serum intact PTH; and hemoglobin concentration. Further analyses were performed to examine the associations between VDRA administration and the other major uremic symptoms. Next, both CHF and intractable edema were extrapolated to the same logistic model as primary outcomes, and the associations between VDRA administration and these outcomes were examined.

### Statistics

Statistical analyses were performed using JMP9.0 (SAS Institute Inc., Cary, NC, USA). Data are expressed as mean ± standard deviation or numbers (percentage) of patients. Comparisons across the various groups were performed using the Pearson Chi-square test for categorical data and the Dunnett test for continuous data. All tests were two-tailed, and a *P* value of <0.05 was considered significant. Factors that were associated with uremic nausea on univariate analysis were subsequently included in a multivariate model. Multivariate logistic regression analyses were performed to identify the covariates that were associated with the development of dialysis-requiring uremic nausea. Odds ratios (ORs) and 95% confidence intervals (CI) were determined using univariate and multivariate logistic regression models for the factors that were significantly associated with uremic nausea. Forrest plots were used to summarize the results of the logistic regression analysis, and Microsoft Excel was used to plot the 95% CI range on the log scale Forrest plots.

## Results

### Prevalence of uremic symptoms at dialysis commencement

Of the 1536 patients, 814 (53%) developed uremic symptoms and started dialysis. Among these patients, 309 (20.1%) developed CHF, 230 (15.0%) developed uremic nausea, 198 (12.9%) developed intractable edema, 77 (5.0%) started dialysis due to other symptoms (anemia, neurological, or other symptoms), and the other 722 (47.0%) started planned dialysis without uremic symptoms.

### Comparison of patients who presented with or without uremic nausea

Compared with the patients who did not develop uremic nausea, the patients who initiated dialysis due to uremic nausea used VDRAs (27.0 vs. 17.4%, *P* = 0.0022) and loop diuretics (71.0 vs. 62.6%, *P* = 0.0108) less frequently (Table 1). However, no significant associations were observed between the prevalence of uremic nausea and the use of ESAs, CaCO_3_, AST-120, CCBs, RAAS inhibitors, and the other anti-hypertensive drugs. The patients who initiated dialysis with uremic nausea also had significantly higher levels of serum creatinine (9.24 ± 3.11 vs. 10.0 ± 3.47 mg/dl, *P* = 0.0005), urea nitrogen (88.6 ± 26.1 vs. 94.2 ± 27.6 mg/dl, *P* = 0.0030), and phosphorus (6.11 ± 1.64 vs. 6.37 ± 1.78 mg/dl, *P* = 0.0259), as well as significantly lower levels of eGFR (5.26 ± 1.94 mL/min/1.73 m^2^ vs. 4.88 ± 1.77 mL/min/1.73 m^2^, *P* = 0.0053) and corrected calcium (8.56 ± 0.94 vs. 8.43 ± 0.88 mg/L, *P* = 0.0452). There were no significant differences between the two groups in age, sex, diabetic kidney disease, medical history of IHD, systolic and diastolic blood pressures, nephrologist follow-up years, hemoglobin, CRP, uric acid, sodium, potassium, chloride, serum albumin, and intact PTH.

**Table 1:** Baseline characteristics and comparison of patients who presented with or without uremic nausea

Variable	Overall (*n* = 1536)	Nausea− (*n* = 1306)	Nausea+ (*n* = 230)	*P* value
Male sex [*n* (%)]	1058 (68.9)	892 (68.3)	166 (72.2)	0.2420
Diabetic kidney disease [*n* (%)]	663 (43.2)	572 (43.8)	91 (39.6)	0.2321
Medical history of IHD [*n* (%)]	288 (18.8)	245 (18.8)	43 (18.7)	0.9817
Age (years)	67.2 ± 13.0	67.2 ± 13.2	67.1 ± 12.1	0.9116
Systolic blood pressure (mmHg)	152.1 ± 24.9	152.3 ± 24.6	151.0 ± 26.3	0.4939
Diastolic blood pressure (mmHg)	77.2 ± 14.4	77.2 ± 14.3	76.8 ± 14.9	0.6577
Nephrologists follow-up (years)	3.69 ± 3.62	3.70 ± 3.75	3.62 ± 2.80	0.7558
*Laboratory data*				
Hemoglobin (g/dl)	8.77 ± 1.48	8.78 ± 1.48	8.67 ± 1.49	0.2995
C-reactive protein (mg/dl)	1.27 ± 3.06	1.34 ± 3.24	0.92 ± 1.70	0.0563
Urea nitrogen (mg/dl)	89.3 ± 26.4	88.6 ± 26.1	94.2 ± 27.6	0.0030
Creatinine (mg/dl)	9.36 ± 3.18	9.24 ± 3.11	10.0 ± 3.47	0.0005
eGFR (mL/min/1.73 m^2^)	5.20 ± 1.92	5.26 ± 1.94	4.88 ± 1.77	0.0053
Uric acid (mg/dl)	8.50 ± 2.23	8.48 ± 2.24	8.58 ± 2.21	0.5636
Sodium (mEq/L)	138.0 ± 4.5	138.1 ± 4.5	137.5 ± 4.2	0.0742
Potassium (mEq/L)	4.60 ± 0.83	4.61 ± 0.83	4.55 ± 0.81	0.2550
Chloride (mEq/L)	105.0 ± 6.0	104.9 ± 6.0	105.3 ± 6.4	0.3366
Corrected calcium (mg/L)	8.54 ± 0.94	8.56 ± 0.94	8.43 ± 0.88	0.0452
Phosphorus (mg/dl)	6.15 ± 1.67	6.11 ± 1.64	6.37 ± 1.78	0.0259
Albumin (g/dl)	3.24 ± 0.59	3.24 ± 0.59	3.25 ± 0.57	0.8696
Intact PTH (pg/ml)	322.6 ± 250.4	320.5 ± 256.7	334.2 ± 211.4	0.5342
CTR (%)	54.1 ± 6.5	54.3 ± 6.6	53.1 ± 6.1	0.0075
*Medications*				
ESA [*n* (%)]	1286 (83.7)	1086 (83.2)	200 (87.0)	0.1498
VDRA [*n* (%)]	392 (25.5)	357 (27.0)	40 (17.4)	0.0022
CaCO_3_ [*n* (%)]	528 (34.4)	452 (34.6)	76 (33.0)	0.6447
Loop diuretics [*n* (%)]	1071 (69.7)	927 (71.0)	144 (62.6)	0.0108
AST-120 [*n* (%)]	308 (20.1)	256 (19.6)	52 (22.6)	0.2936
CCB [*n* (%)]	1166 (75.9)	990 (75.8)	176 (76.5)	0.8144
RAAS inhibitors	1016 (66.2)	867 (65.6)	159 (69.1)	0.2996
Other hypertensive drugs	701 (45.6)	598 (45.8)	103 (44.8)	0.7776

Data are expressed as numbers (%) of patients or mean ± SD

*IHD* ischemic heart disease, *eGFR* estimated glomerular filtration rate, *PTH* parathyroid hormone, *CTR* cardiothoracic ratio, *ESA* erythropoiesis-stimulating agent, *VDRA* vitamin D receptor activator, *CaCO*_*3*_ calcium carbonate, *AST-120* an orally administered uremic toxin adsorbent, *CCB* calcium channel blocker; *RAAS* renin-angiotensin-aldosterone system

### Associations between various risk factors and the prevalence of uremic nausea

As shown in Table 2, univariate and multivariate-adjusted logistic regression analyses were used to assess the independent associations between uremic nausea and medications or the other clinical parameters. The primary outcome was the prevalence of uremic nausea at dialysis initiation, the predictor variable was VDRA use during the predialysis period, and the covariates were determined according to their univariate relationship or importance within the explanatory variables.

**Table 2:** Association between various factors and the prevalence of uremic nausea

Variable	Univariable model		Multivariable model	
	OR (95% CI)	*P* value	OR (95% CI)	*P* value
Age (years)	0.999 (0.989–1.010)	0.9116	1.007 (0.995–1.020)	0.2381
Diabetic kidney disease	0.840 (0.630–1.116)	0.2306	0.896 (0.656–1.221)	0.4872
Nephrologists follow-up	0.994 (0.954–1.032)	0.7543	0.994 (0.952–1.035)	0.7736
Male sex	0.996 (0.688–1.414)	0.9817	1.216 (0.868–1.719)	0.2575
CTR	0.970 (0.948–0.992)	0.0068	0.967 (0.943–0.991)	0.0069
*Laboratory data*				
Hemoglobin	0.951 (0.864–1.046)	0.2988	0.943 (0.853–1.041)	0.2470
eGFR (mL/min/1.73 m^2^)	0.892 (0.822–0.965)	0.0040	0.907 (0.821–0.998)	0.0458
Corrected calcium (mg/L)	0.860 (0.742–0.997)	0.0462	0.895 (0.763–1.050)	0.1741
Phosphorus (mg/dl)	1.096 (1.010–1.189)	0.0279	1.058 (0.960–1.165)	0.2564
Intact PTH	1.000 (1.000–1.001)	0.4535	1.000 (0.999–1.001)	0.8936
*Medications*				
ESAs	1.351 (0.909–2.070)	0.1401	1.438 (0.958–2.225)	0.0810
VDRAs	0.571 (0.392–0.811)	0.0015	0.512 (0.347–0.738)	0.0003
CaCO_3_	0.932 (0.690–1.251)	0.6439	0.923 (0.669–1.265)	0.6216
Loop diuretics	0.685 (0.512–0.920)	0.0121	0.733 (0.536–1.006)	0.0547

*CTR* cardiothoracic ratio, *eGFR* estimated glomerular filtration rate, *PTH* parathyroid hormone, *ESA* erythropoiesis-stimulating agent, *VDRA* vitamin D receptor activator, *CaCO*_*3*_ calcium carbonate

Although lower levels of corrected calcium and higher levels of phosphorus were risk factors for uremic nausea on univariate analyses, they were not independent risk factors on multivariate-adjusted logistic regression analysis. Loop diuretic use was negatively associated with uremic nausea on univariate analysis, but its association also disappeared on multivariate analysis. No significant associations were observed between uremic nausea and the prevalence of diabetic kidney disease, nephrologist follow-up period, and intact PTH.

The multivariate-adjusted logistic regression analysis showed that, of all the medications examined, only VDRA use (OR 0.512, 95% CI 0.347–0.738, *P* = 0.0003) was independently associated with a significantly reduced risk of dialysis-requiring uremic nausea, adjusted by chronic kidney disease-mineral and bone disorder (CKD-MBD)-associated factors, such as corrected calcium, phosphorus, intact PTH, CaCO_3_ (medication), and nephrologist follow-up period. In addition, eGFR (OR 0.907, 95% CI 0.821–0.998, *P* = 0.0458) and the cardiothoracic ratio (CTR) (OR 0.967, 95% CI 0.943–0.991, *P* = 0.0069) were independently associated with uremic nausea on logistic regression analysis. On the other hand, no significant association was observed between uremic nausea and CaCO_3_ administration on logistic regression analysis.

### Comparison of patients’ characteristics between VDRA users and non-users

Since only VDRA was a possible candidate medication for reducing the risk of uremic nausea, the characteristics of VDRA users and VDRA non-users (*n* = 392 vs 1144 patients, 25.5 vs 74.5%) were compared to confirm whether there were any significant differences between the two groups. As shown in Table 3, compared with the VDRA non-users, the VDRA users were significantly younger (67.8 ± 12.9 vs. 65.4 ± 13.2 years, *P* = 0.0020), had a lower proportion of males (70.4 vs. 64.5%, *P* = 0.0315), had a lower frequency of diabetic kidney disease (46.3 vs. 33.9%, *P* < 0.0001), had a lower frequency of IHD (20.6 vs. 13.3%, *P* = 0.0013), had a longer nephrologist follow-up period (3.45 ± 3.48 vs. 4.40 ± 3.92 years, *P* < 0.0001), had higher frequencies of ESA use (80.9 vs. 91.8%, *P* < 0.0001), CaCO_3_ use (29.8 vs. 47.7%, *P* < 0.0001), and loop diuretic use (63.5 vs. 71.9%, *P* < 0.0019), and had significantly higher levels of serum creatinine (9.21 ± 3.12 vs. 9.80 ± 3.31 mg/dl, *P* = 0.0015) and albumin (3.17 ± 0.59 vs. 3.44 ± 0.53 g/dl, *P* < 0.0001). VDRA users also had a lower frequency of uremic nausea compared with VDRA non-users (16.6 vs. 10.2%, *P* = 0.0022), despite their lower levels of eGFR, which are usually associated with a higher prevalence of uremic nausea.

**Table 3:** Comparison of patients’ characteristics between VDRA users and non-users

Variable	VDRA− (*n* = 1144)	VDRA+ (*n* = 392)	*P* value
Male sex [*n* (%)]	805 (70.4)	253 (64.5)	0.0315
Diabetes kidney disease [*n* (%)]	530 (46.3)	133 (33.9)	<0.0001
Medical history of IHD [*n* (%)]	236 (20.6)	52 (13.3)	0.0013
Age (years)	67.8 ± 12.9	65.4 ± 13.2	0.0020
Systolic blood pressure (mmHg)	152.6 ± 24.8	150.6 ± 25.1	0.1737
Diastolic blood pressure (mmHg)	77.0 ± 14.6	77.7 ± 14.6	0.3460
Nephrologist follow-up (years)	3.45 ± 3.48	4.40 ± 3.92	<0.0001
*Laboratory data*			
Hemoglobin (g/dl)	8.74 ± 1.46	8.85 ± 1.52	0.1982
C-reactive protein (mg/dl)	1.31 ± 2.93	1.16 ± 3.42	0.3810
Urea nitrogen (mg/dl)	89.0 ± 26.6	90.6 ± 25.8	0.2839
Creatinine (mg/dl)	9.21 ± 3.12	9.80 ± 3.31	0.0015
eGFR (mL/min/1.73 m^2^)	5.31 ± 1.97	4.90 ± 1.74	0.0003
Uric acid (mg/dl)	8.59 ± 2.27	8.22 ± 2.12	0.0045
Sodium (mEq/L)	138.1 ± 4.5	137.7 ± 4.3	0.0951
Potassium (mEq/L)	4.59 ± 0.83	4.64 ± 0.83	0.2573
Chloride (mEq/L)	105.0 ± 6.1	104.9 ± 5.8	0.7838
Corrected calcium (mg/L)	8.55 ± 0.93	8.52 ± 0.94	0.6670
Phosphorus (mg/dl)	6.16 ± 1.65	6.14 ± 1.71	0.8495
Albumin (g/dl)	3.17 ± 0.59	3.44 ± 0.53	<0.0001
Intact PTH (pg/ml)	329.0 ± 246.7	303.7 ± 260.3	0.0834
CTR (%)	54.3 ± 6.6	53.1 ± 6.1	0.0075
*Medications*			
ESA [*n* (%)]	926 (80.9)	360 (91.8)	<0.0001
CaCO_3_ [*n* (%)]	341 (29.8)	187 (47.7)	<0.0001
Loop diuretics [*n* (%)]	822 (71.9)	249 (63.5)	0.0019
AST-120 [*n* (%)]	235 (20.5)	73 (18.6)	0.4127
CCB [*n* (%)]	859 (75.1)	307 (78.3)	0.1970
RAAS inhibitors	762 (66.6)	254 (64.8)	0.5128
Other anti-hypertensive drugs	523 (45.7)	178 (45.4)	0.9157

Data are expressed as numbers (%) of patients or mean ± SD

*IHD* ischemic heart disease, *eGFR* estimated glomerular filtration rate, *PTH* parathyroid hormone, *CTR* cardiothoracic ratio, *ESA* erythropoiesis-stimulating agent, *VDRA* vitamin D receptor activator, *CaCO*_*3*_ calcium carbonate, *AST-120* an orally administered uremic toxin adsorbent, *CCB* calcium channel blocker, *RAAS* renin-angiotensin-aldosterone system

On the other hand, no significant differences were observed in serum corrected calcium, phosphorus, and intact PTH between VDRA users and non-users. This may reflect successful adjustment of hypocalcemia and secondary hyperparathyroidism by VDRA use in VDRA users.

### Association between VDRA administration during the predialysis period and dialysis-requiring CHF development

Further analyses were performed to examine the associations between VDRA administration and the other major uremic symptoms. First, dialysis-requiring CHF development was extrapolated to the same logistic model, and the association between VDRA administration and CHF was examined. The primary outcome was the prevalence of CHF at dialysis initiation, the predictor variable was VDRA use, and the covariates were the same as those used in the analysis for uremic nausea described above (Table 4).

**Table 4:** Association between various risk factors and the prevalence of CHF

Variable	Univariable model		Multivariable model	
	OR (95% CI)	*P* value	OR (95% CI)	*P* value
Age (years)	1.013 (1.003–1.023)	0.0085	1.001 (0.989–1.013)	0.8864
Diabetic kidney disease	1.851 (1.440–2.383)	<0.0001	1.653 (1.239–2.208)	0.0006
Nephrologist follow-up	0.941 (0.902–0.978)	0.0017	0.976 (0.935–1.017)	0.2532
Male sex	1.062 (0.812–1.398)	0.6632	0.765 (0.552–1.052)	0.1004
CTR	1.121 (1.099–1.145)	<0.0001	1.120 (1.094–1.146)	<.0001
*Laboratory data*				
Hemoglobin	0.876 (0.803–0.954)	0.0022	0.908 (0.827–0.998)	0.0446
eGFR (mL/min/1.73 m^2^)	1.219 (1.147–1.296)	<0.0001	1.187 (1.097–1.285)	<0.0001
Corrected calcium (mg/L)	1.210 (1.056–1.389)	0.0058	1.212 (1.034–1.422)	0.0173
Phosphorus (mg/dl)	0.995 (0.923–1.072)	0.8985	1.105 (1.002–1.218)	0.0446
Intact PTH	0.9998 (0.999–1.000)	0.5273	1.000 (0.999–1.001)	0.6979
*Medications*				
ESAs	0.825 (0.599–1.151)	0.2535	0.891 (0.627–1.281)	0.5281
VDRAs	0.572 (0.413–0.780)	0.0003	0.817 (0.573–1.152)	0.2524
CaCO_3_	0.645 (0.487–0.848)	0.0016	0.987 (0.704–1.394)	0.2432
Loop diuretics	2.378 (1.746–3.292)	<0.0001	1.547 (1.100–2.202)	0.0118

*CTR* cardiothoracic ratio, *eGFR* estimated glomerular filtration rate, *PTH* parathyroid hormone, *ESA* erythropoiesis-stimulating agent, *VDRA* vitamin D receptor activator, *CaCO*_*3*_ calcium carbonate

Although VDRA and CaCO_3_ use, older age, and shorter nephrologist follow-up were associated with CHF development on univariate analyses, multivariate analysis showed that VDRA administration in the predialysis period was not independently associated with dialysis-requiring CHF development (OR 0.817; 95% CI 0.573–1.152; *P* = 0.2524).

### Association between VDRA administration during the predialysis period and intractable edema

As shown in Table 5, the same univariate and multivariate-adjusted logistic regression models were used to assess the independent association between intractable edema and VDRA use. The multivariate analysis also showed that VDRA administration in the predialysis period was not independently associated with intractable edema development at dialysis commencement (OR 0.766; 95% CI 0.517–1.115; *P* = 0.1662).

**Table 5:** Associations between various risk factors and the prevalence of intractable edema

Variable	Univariable model		Multivariable model	
	OR (95% CI)	*P* value	OR (95% CI)	*P* value
Age (years)	0.998 (0.987–1.010)	0.7820	0.996 (0.983–1.009)	0.5668
Diabetic kidney disease	1.472 (1.091–1.986)	0.0114	1.193 (0.862–1.651)	0.2881
Nephrologists follow-up	0.985 (0.941–1.026)	0.4809	1.005 (0.960–1.048)	0.8323
Male sex	0.743 (0.546–1.018)	0.0646	0.656 (0.465–0.930)	0.0180
CTR	1.023 (1.000–1.046)	0.0455	1.009 (0.984–1.034)	0.4956
*Laboratory data*				
Hemoglobin	0.944 (0.853–1.044)	0.2655	0.966 (0.868–1.074)	0.5178
eGFR (mL/min/1.73 m^2^)	1.063 (0.986–1.144)	0.1096	1.078 (0.985–1.177)	0.1005
Corrected calcium (mg/L)	1.046 (0.892–1.230)	0.5780	1.020 (0.854–1.219)	0.8251
Phosphorus (mg/dl)	1.046 (0.957–1.141)	0.3200	1.100 (0.988–1.223)	0.0823
Intact PTH	0.999 (0.999–1.000)	0.1091	0.999 (0.999–1.000)	0.1226
*Medications*				
ESAs	1.473 (0.957–2.364)	0.0795	1.535 (0.985–2.487)	0.0584
VDRAs	0.734 (0.504–1.048)	0.0896	0.766 (0.517–1.115)	0.1662
CaCO_3_	0.948 (0.688–1.296)	0.7404	0.987 (0.704–1.394)	0.9398
Loop diuretics	2.945 (1.975–4.549)	<0.0001	2.571 (1.692–4.034)	<0.0001

*CTR* cardiothoracic ratio, *eGFR* estimated glomerular filtration rate, *PTH* parathyroid hormone, *ESA* erythropoiesis-stimulating agent, *VDRA* vitamin D receptor activator, *CaCO*_*3*_ calcium carbonate

### Associations between VDRA administration and the prevalence of major uremic symptoms at dialysis commencement

**Fig. 2:**
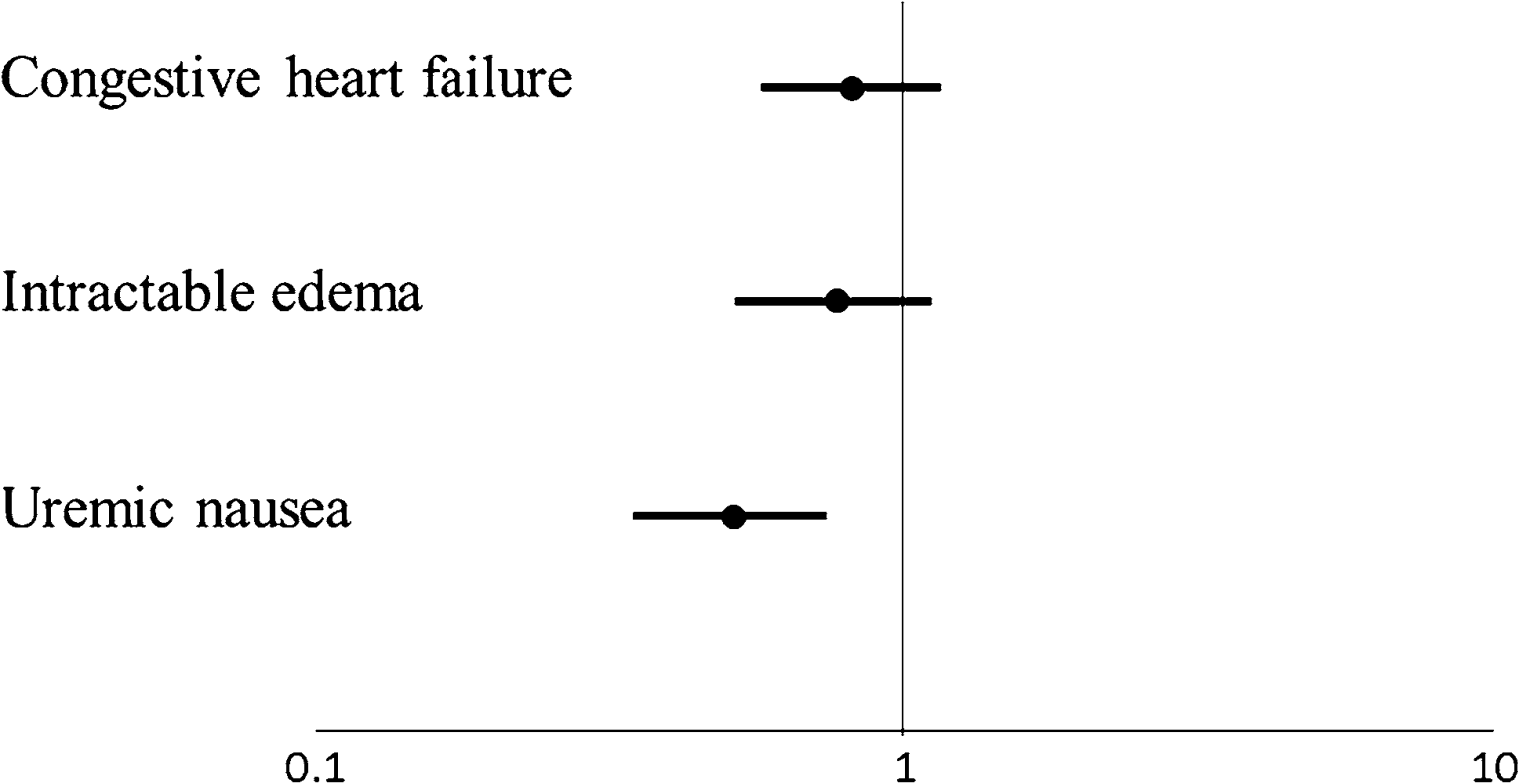
Associations of VDRA administration with the prevalence of major uremic symptoms at dialysis commencement. Log scale Forrest plots summarize the association between use of VDRA in the predialysis period and the prevalence of each uremic symptom just prior to initiation of dialysis. Use of VDRAs in the predialysis period is significantly negatively associated with uremic nausea development. On the other hand, use of VDRAs is not associated with the other major uremic symptoms, congestive heart failure, and intractable edema, at dialysis commencement

Log scale Forrest plots show the summary of the multivariate-adjusted logistic regression analysis (Fig. 2). Use of VDRAs in the predialysis period was significantly negatively associated with uremic nausea development. On the other hand, use of VDRAs was not associated with the other major uremic symptoms, CHF and intractable edema, at dialysis commencement.

## Discussion

In this retrospective cross-sectional study, only VDRA administration was found to be independently associated with a significantly reduced risk of either uremic nausea or all uremic symptom development, adjusted by CKD-MBD-associated factors, such as corrected calcium, phosphorus, intact PTH, and CaCO_3_ administration, and nephrologist follow-up period.

On the other hand, VDRA use during the predialysis period was not significantly associated with the other major uremic symptoms, CHF, and intractable edema, in the same multivariate model. These results suggest that the strong association between VDRA use and uremic nausea may cause a significant association between all uremic symptoms and VDRA use. To the best of our knowledge, this is the first paper to identify a negative association between VDRA administration and uremic nausea in patients with advanced CKD.

In the present cohort, VDRA users had a significantly lower prevalence of uremic nausea than VDRA non-users (10.2 vs 16.6%, *P* = 0.0022), despite their significantly lower level of eGFR at the initiation of dialysis (4.90 ± 1.74 mL/min/1.73 m^2^ vs 5.31 ± 1.97 mL/min/1.73 m^2^, *P* = 0.0003). Interestingly, there were no significant differences in serum corrected calcium, phosphorus, and intact PTH between VDRA users and non-users, suggesting that VDRA use might successfully correct hypocalcemia (VDRA users vs non-users: 8.52 ± 0.94 vs 8.55 ± 0.93 mg/L, *P* = 0.6670).

There were several confounding factors in the analysis comparing VDRA users and non-users on the univariate analysis: VDRA users were significantly younger, had a higher proportion of female sex, lower frequency of diabetic kidney disease, longer nephrologist follow-up period, higher frequency of ESA use and CaCO_3_ use, and lower frequency of loop diuretic use than VDRA non-users. However, only VDRA administration was significantly associated with a lower prevalence of uremic nausea at dialysis commencement on multivariate-adjusted logistic regression analysis after adjustment for these confounding factors (OR 0.512; 95% CI 0.347–0.738; *P* = 0.0003). The significant independent relationship between VDRA use and uremic nausea was also confirmed even after addition of creatinine instead of eGFR to the logistic regression analysis (OR 0.513; 95% CI 0.348–0.740; *P* = 0.0003). These results suggest that the potential ability of VDRA to prevent uremic nausea resulted in this association.

In addition, both oral calcium carbonate administration and the other CKD-MBD-related factors (corrected calcium, phosphorus, and intact PTH use) were not associated with uremic nausea in the present logistic regression analysis, suggesting that VDRAs might have an action on uremic nausea that differs from regulation of calcium homeostasis and bone turnover.

In the present cross-sectional study, why VDRA use was associated with uremic nausea is unknown. VDRAs may reduce uremic nausea via the anti-inflammatory system by blocking NF-κB activation in patients with CKD [7–10]. There was a small, but not significant, decrease in the serum CRP level in VDRA users compared with non-users in the present cohort (1.16 ± 3.42 vs 1.31 ± 2.93 mg/dl, *P* = 0.3810). This decrease in CRP might affect uremic nausea development. Thus, one cannot rule out the possibility that VDRA use might reduce uremic nausea via anti-inflammatory actions.

Several studies have suggested that VDRAs may interfere with uremic nausea via GI tract cells and/or neuronal cells that constitute the nausea/vomiting reflex pathway. Ubiquitous expression of VDR has been reported, including by both gastrointestinal tract cells [8] and neuronal cells. For example, VDR and 25 hydroxyvitamin D3-24 hydroxylase (24OHase) mRNA have been shown to be expressed in the central nervous system [13].

Uremic anorexia in chronic dialysis patients is a complex complication associated with malnutrition and high levels of morbidity and mortality. Factors associated with uremic anorexia include high cerebrospinal fluid levels of proinflammatory cytokines, leptin, and free tryptophan and serotonin, along with a deficiency of neural nitric oxide (nNO) and disorders in various receptors, such as melanocortin receptor-4 (MC4-R) [4, 14, 15]. Gastric hypomotility has been reported as a possible cause of uremic nausea in predialysis patients [16]. Abnormalities in body fluid volume, serum electrolyte concentrations, and acid–base balance and accumulations of uremic toxic substances impair gastric emptying. Delayed gastric emptying is also frequent in dialysis patients, even those who do not have diabetes mellitus [17].

On the other hand, uremic nausea in predialysis patients may be a functional disease without pathological organic abnormalities, because it improves soon after the first dialysis session. Once toxic uremic substances are removed or corrected by the first dialysis session, uremic nausea usually resolves.

Since metabolic acidosis in CKD patients may worsen uremic nausea, the association between serum bicarbonate concentration and uremic nausea was further analyzed in 1387 patients. A significant difference in serum bicarbonate concentration was seen between patients with and without uremic nausea on univariate analysis (uremic nausea present: 18.1 ± 4.7 mEq/L, *n* = 201 vs uremic nausea absent: 18.9 ± 4.7 mEq/L, *n* = 1186, *P* = 0.0197). Another logistic regression analysis was performed by adding serum bicarbonate concentration as an explanatory variable; VDRA use was independently associated with uremic nausea (OR 0.510, 95% CI 0.338–0.754, *P* = 0.0006), but serum bicarbonate concentration was not (OR 0.983, 95%CI 0.947–1.019, *P* = 0.3493).

VDRAs may interfere with uremic substances and improve gastrointestinal and neuronal dysfunction.

We have reported that VDRA administration was not independently associated with the CHF development at dialysis commencement [11]. In this study, VDRA administration in the predialysis period was negatively associated with uremic nausea that needed dialysis commencement. Taken together, VDRA administration in the predialysis period has different associations with either uremic nausea or CHF development.

This study also demonstrated an independent association between CTR and uremic nausea on logistic regression analysis. Nausea usually causes volume depletion via a decrease in oral intake, so that the CTR may tend to decrease in patients with uremic nausea. In this study, patients with uremic nausea showed a significant decrease in the CTR compared to patients with CHF or intractable edema (53.1 ± 6.1% vs. 58.0 ± 6.7%, *P* < 0.0001, 55.0 ± 6.6%, *P* = 0.0049).

If VDRAs effectively prevent uremic nausea, the patients’ nutritional status may improve. In fact, serum albumin concentrations of VDRA users were significantly higher than those of non-users (3.44 ± 0.53 vs 3.17 ± 0.59 g/dl, *P* < 0.0001), suggesting that VDRAs may prevent uremic nausea, improve nutritional status, and finally prevent early initiation of dialysis. Of course, volume overload in patients with CHF or intractable edema might cause dilution of the serum albumin concentration.

There are several limitations in this study. First, this study used a cross-sectional design, there was no information regarding the types, dosage, and duration of each medication, and the serum vitamin D concentration was not known. Second, there were no data from either endoscopy or gastric motility testing, so that the associations between these pieces of gastrointestinal information and uremic nausea remain unknown. Third, there were also no data about the concentrations of the serum appetite regulators described above, so that how VDRA could prevent uremic nausea via interacting with these appetite regulators could not be examined. Fourth, the patients’ lipid profiles or use of statins and other lipid-lowering drugs was not analyzed, since such information was not consistently available. These factors may also contribute to the present results. Therefore, additional studies are needed to clarify the effects of medications on uremic nausea development in patients with stage 5 CKD. This study’s results suggest that VDRAs may decrease uremic nausea, but whether their use decreases mortality and morbidity is not known. Without randomized clinical trials, causation cannot be inferred from observational designs.

## Conclusion

VDRA administration in the predialysis period was found to be associated with a lower prevalence of uremic nausea, suggesting that VDRA use in patients with advanced CKD may prevent uremic nausea. However, well-controlled prospective studies are needed to confirm these results, given the present study’s cross-sectional design.
